# Optimizing the Real-World Use of BCMA-Targeted T-Cell-Engaging Therapies in Patients with Triple-Class-Exposed Relapsed/Refractory Multiple Myeloma: An Italian Modified Delphi Consensus

**DOI:** 10.3390/cancers18162572

**Published:** 2026-08-10

**Authors:** Michele Cavo, Melissa Bersanelli, Alessandro Corso, Carlotta Galeone, Silvia Mangiacavalli, Roberto Mina, Renato Zambello, Elisabetta Antonioli, Angelo Belotti, Cirino Botta, Gabriele Buda, Francesco Di Raimondo, Monica Galli, Francesca Gay, Massimo Offidani, Maria Teresa Petrucci, Alessandra Romano, Elena Zamagni, Antonella Semeraro, Barbara Veggia, Paolo Mariani

**Affiliations:** 1 Department of Medical and Surgical Sciences, University of Bologna, Via Massarenti 9, 40138 Bologna, Italy; 2 Pfizer Italia, 00188 Rome, Italy; melissa.bersanelli@pfizer.com (M.B.); barbara.veggia@pfizer.com (B.V.); 3 Hematology and Stem Cell Transplantation Division, Hospital Legnano, 20025 Legnano, Italy; 4 Bicocca-Applied Statistics Center (B-ASC), Department of Economics, Management and Statistics, University of Milan-Bicocca, Via Bicocca degli Arcimboldi 8, 20126 Milan, Italy; 5 U.O.C. Ematologia 1, IRCCS Fondazione Policlinico San Matteo, 27100 Pavia, Italy; 6 Division of Hematology, Department of Molecular Biotechnology and Health Sciences, AOU Città della Salute e della Scienza di Torino, University of Turin, 10126 Turin, Italy; 7 Dipartimento di Medicina (DIMED), Ematologia e Immunologia Clinic, Università di Padova, 35128 Padova, Italy; r.zambello@unipd.it; 8 Haematology Unit, Careggi University Hospital, 50134 Florence, Italy; 9 Department of Hematology, ASST Spedali Civili di Brescia, 25123 Brescia, Italy; 10 Department of Health Promotion, Mother and Child Care, Internal Medicine and Medical Specialties “G. D’Alessandro”, University of Palermo, 90127 Palermo, Italy; 11 Ematologia Univ, Dipartimento di Medicina Clinica e Sperimentale, Università di Pisa, 56126 Pisa, Italy; g.buda@ao-pisa.toscana.it; 12 A.O.U. Policlinico “G. Rodolico-San Marco”, 95123 Catania, Italy; 13 Dipartimento di Specialità Medico-Chirurgiche, CHIRMED, Sezione di Ematologia, Università degli Studi di Catania, 95131 Catania, Italy; 14 Hematology and Bone Marrow Transplant Unit, ASST Papa Giovanni XXIII, 24127 Bergamo, Italy; 15 Division of Hematology 1, Azienda Ospedaliero-Universitaria Città della Salute e della Scienza di Torino, Department of Molecular Biotechnology and Health Sciences, University of Torino, 10126 Torino, Italy; 16 Clinica di Ematologia, AOU delle Marche, 60126 Ancona, Italy; 17 Hematology, Azienda Ospedaliera Universitaria Policlinico Umberto I, Sapienza University of Rome, 00161 Roma, Italy; 18 Istituto di Ematologia “Seràgnoli”, IRCCS Azienda Ospedaliero-Universitaria di Bologna, 40138 Bologna, Italy

**Keywords:** B-cell maturation antigen, bispecific antibodies, Delphi consensus, multiple myeloma, treatment, triple-class-exposed patients

## Abstract

Background/Objectives: The treatment landscape of relapsed/refractory multiple myeloma (RRMM) has significantly evolved with the introduction of novel classes of agents and, more recently, of T-cell-redirecting therapies, including bispecific antibodies (BsAbs). It is essential to enhance and harmonize the therapeutic management of BsAbs within clinical practice. Methods: We performed a modified Delphi expert consensus study on the use of BsAbs targeting the B-Cell Maturation Antigen (BCMA) in patients with triple-class-exposed (TCE) RRMM. The study was conducted in April–November 2025 following established guidelines and best practices for defining consensus. The key phases in the use of anti-BCMA BsAbs were identified and explored. Results: Fifteen Italian hematologists with expertise in the care of TCE RRMM completed two Delphi rounds. Agreement (defined as ≥67% of panelists) was achieved on most of the topics evaluated. In particular, all panelists considered the step-up dosing phase feasible in an outpatient setting, under specific circumstances, and dosing de-escalation in responding patients to reduce the risk of adverse events. For most of them, anti-BCMA BsAb treatment is also feasible in several challenging subgroups, including frail patients (93% agreement) and those with high-risk cytogenetics (93%), extramedullary disease (93%), end-stage renal disease (86%), active plasma cell leukemia (79%), and central nervous system involvement (67%). In addition, agreement (93%) was reached on the possible sequential use of BCMA-targeting therapies, preferentially BsAbs following CAR-T, though a switch in the target antigen should primarily be considered. Conclusions: In this article, we address the main challenges related to the real-world use of anti-BCMA BsAbs in patients with TCE RRMM, offering expert recommendations to complement existing guidelines and support clinical practice.

## Simple Summary

Bispecific antibodies are newly available medicines for multiple myeloma. They help the immune system recognize and attack myeloma cells, but their use in daily practice can be challenging, especially in patients with complex clinical conditions. This study collected the views of Italian myeloma experts on how to use bispecific antibodies targeting B-cell maturation antigen in patients whose disease had relapsed or become refractory after using the main available treatment classes. Using a structured consensus process, experts agreed on several practical aspects, including outpatient treatment under specific circumstances, reducing dosing in responding patients, and use in frail patients or in those with high-risk disease or organ involvement. These recommendations may support more consistent and safer clinical decisions on the use of bispecific antibodies targeting B-cell maturation antigen and help identify questions for future research.

## 1. Introduction

Multiple myeloma (MM) is a B-cell lymphoid neoplasm characterized by the proliferation of clonal plasma cells in the bone marrow [1]. Although formally classified as a rare disease according to Orphanet criteria (ORPHA: 29073), it ranks third among the most frequent hematological cancers, and an increasing incidence in most areas of the world has been reported over the past 30 years [2,3,4].

During the past two decades, three major classes of novel agents, namely immunomodulatory drugs (IMiDs), proteasome inhibitors (PIs), and anti-CD38 monoclonal antibodies (mAbs), have progressively become available and led to substantial improvements in patient survival [5]. Nevertheless, multiple relapses continue to occur during the natural history of the disease, and the management of relapsed/refractory MM (RRMM) in late lines of therapy is increasingly challenging. Historically, triple-class-exposed patients (i.e., those exposed to an IMiD, a PI, and an anti-CD38 mAb) with RRMM (TCE RRMM) had very poor outcomes due to limited salvage treatments and have represented a clinical need requiring the identification of novel agents with new mechanisms of action [6]. T-cell-redirecting immunotherapies use either chimeric antigen receptor (CAR) T cells or bispecific T-cell-engaging antibodies (BsAbs), targeting an antigen expressed on the surface of MM cells, most frequently the B-cell maturation antigen (BCMA) or G-protein-coupled receptor class C group 5 member D (GPRC5D); these showed remarkable efficacy in heavily pre-treated RRMM and received approval by the FDA and EMA for the management of patients with TCE RRMM who were refractory to their last therapy after at least 3 (EMA label) or 4 (FDA label) prior lines of treatment [7,8,9]. Accordingly, the term TCE is used throughout this manuscript to ensure consistency with the regulatory eligibility framework applicable to BCMA-directed CAR-T-cell therapies and BsAbs during the study period.

Often referred to as off-the-shelf alternatives to CAR-T-cell therapies [9,10], BsAbs simultaneously bind to CD3 on the T-cell surface and to the target antigen, thus promoting the formation of an immune synapse with the subsequent activation of T cells, which release granzymes and perforins, ultimately leading to tumor cell lysis. The paradigm shift introduced by the novel immunotherapies engaging T cells lies in their ability to enhance the intrinsic activity of the immune system, thus at least partly overcoming the immune evasion mechanisms typical of MM [11,12].

BsAbs have been introduced into the therapeutic armamentarium for MM quite recently, and educational efforts to guide their expanded use and safe management in daily clinical practice are needed [13]. Furthermore, data from real-world studies are currently limited, specifically at a national level [14,15,16,17,18]. This study aimed to collect clinical practice recommendations on the optimal use of BsAbs for TCE RRMM patients provided by Italian experts in this field. For this purpose, their advice, particularly focused on anti-BCMA T-cell-engaging BsAbs, was collected using a modified Delphi consensus methodology.

## 2. Methods

This modified Delphi study followed established guidelines and best practices for defining consensus, including participant anonymity, repeated rounds, controlled feedback, and statistical analysis of agreement [19]. The entire investigation was documented in accordance with the ACcurate COnsensus Reporting Document (ACCORD) guidelines [20].

**Figure 1:**
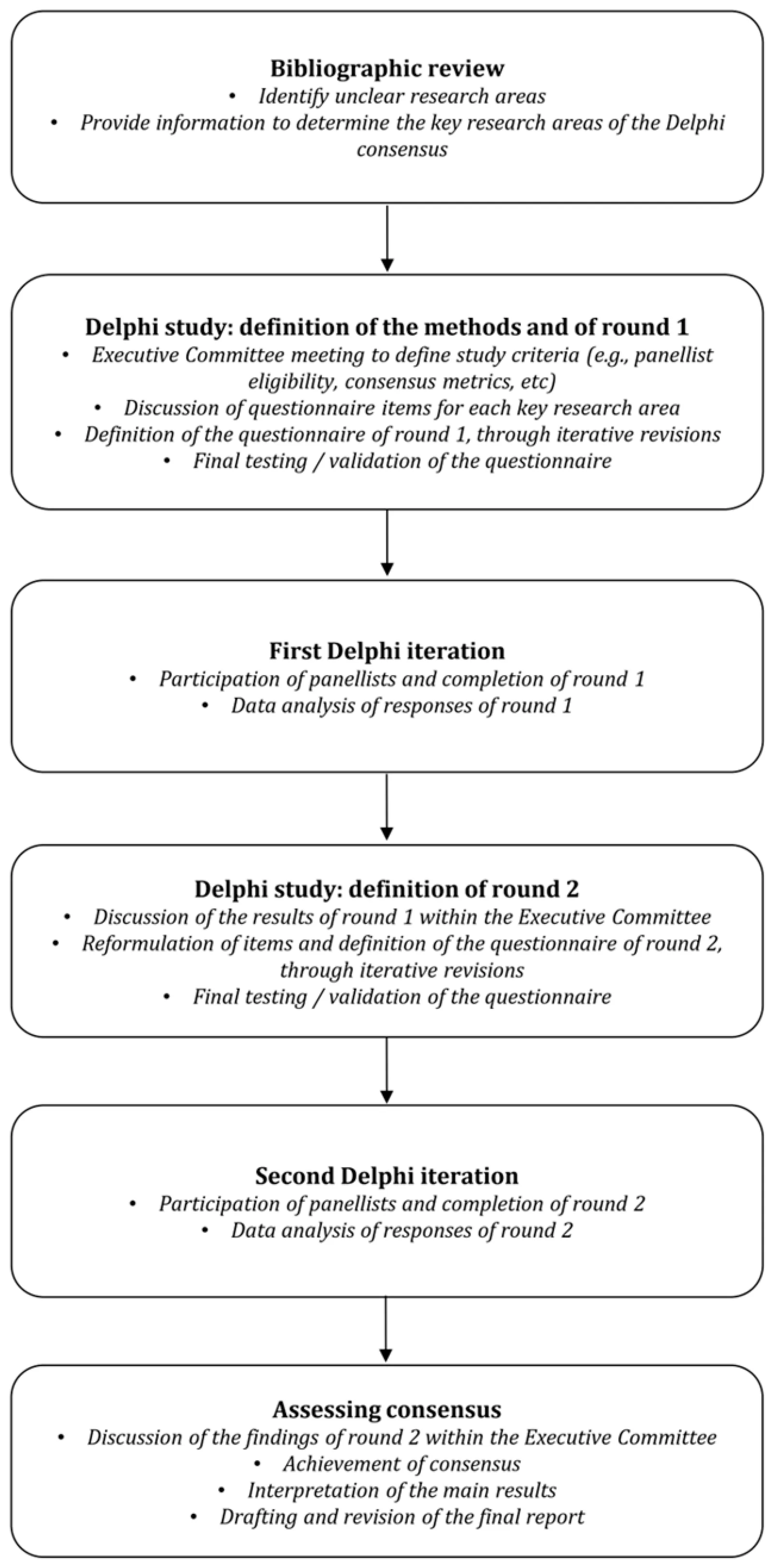
Flowchart reporting Delphi consensus phases.

The study was conceived in late 2024 and was practically conducted between April and November 2025, involving MM specialists working in various Italian regions. The phases and methodologies of the investigation are outlined in Figure 1 and described thoroughly below. Additional details on the methodology used (e.g., on search strings, Executive Committee meetings, and selected aspects related to consensus) are provided in the Supplementary Materials. The Delphi consensus protocol was not pre-registered on any platform.

### 2.1. Preliminary Bibliographic Review

In April and May 2025, we conducted an extensive, narrative literature review of studies focused on the use of anti-BCMA BsAbs in TCE RRMM patients, in order to identify unaddressed or non-completely addressed topics and clinical practice areas to be investigated in the Delphi consensus survey. The bibliographic review started on 5 April 2025 (i.e., the date of the literature search) and was conducted using PubMed/Medline, EMBASE, and Google Scholar. Identified articles were screened, and relevant ones were selected for further consideration. In the synthesis of findings, specific relevance was given to evidence-based guidelines, position papers, expert recommendations, and real-world studies. The main results were summarized and discussed within the Executive Committee, and six key topics were outlined for further investigation during the Delphi process.

The key areas were as follows: (1) the initial choice of T-cell-engaging immunotherapy, particularly anti-BCMA BsAbs; (2) a workup assessment before starting anti-BCMA BsAbs; (3) an anti-BCMA BsAb step-up dosing (SUD) phase and its inpatient or outpatient management; (4) patient monitoring during anti-BCMA BsAb treatment; (5) the prevention and management of adverse events related to anti-BCMA BsAb treatment; (6) the feasibility of treatment in special patient populations (i.e., frail patients according to different scores/characteristics, patients with high-risk cytogenetics, those with active plasma cell leukemia [PCL], those with central nervous system [CNS] involvement, those with end-stage renal disease [ESRD; defined as renal failure requiring dialysis or transplantation, or an eGFR <15 mL/min/1.73 m^2^ for at least 4 weeks], and those with extramedullary disease). At the time the Delphi survey was designed and conducted, cilta-cel had not yet received approval by the Italian Medicines Agency (AIFA) for use in patients with TCE RRMM in Italy. Consequently, cilta-cel was not included among the treatment options available in clinical practice during the study period.

The questionnaire of the first Delphi iteration included a number of pertinent items for each key area of research. The questionnaire was revised multiple times by the Executive Committee group, until a final version was defined. The latter was tested in a preliminary phase by one of the Executive Committee members before starting the voting phases. The questionnaire for the second Delphi iteration was developed iteratively based on the aggregated responses and free-text comments collected during round 1. It included reformulated items for a few topics that did not achieve consensus, and new questions addressing clinically relevant issues that emerged during the first round. More specifically, after reviewing the findings of the first round regarding the most frequent types of adverse events associated with the use of BsAbs, a detailed section on the management of each common type of adverse event was included in the second round through a structured set of follow-up questions addressing the timing of occurrence, prophylaxis, signs and symptoms, diagnostic workup, treatment, multidisciplinary management, training needs, and potential differences across BsAbs.

### 2.2. Executive Committee

An Executive Committee formed by a biostatistician/epidemiologist (C.G.), a Delphi Master (P.M.), and an expert hematologist (M.C.) contributed to design the Delphi study, to define the questionnaires, and to identify the inclusion criteria of the panelists, as well as the consensus criteria. The Executive Committee interpreted the results of Delphi iterations and drafted the final manuscript, which was approved by all the authors. Executive Committee members had established expertise in the conduct and publication of Delphi studies in the biomedical field [21].

Members of the Executive Committee were not involved in the panel group and thus did not vote in any Delphi iteration.

### 2.3. Characteristics of the Survey and Criteria for Consensus

The goal of the Delphi rounds was to reach consensus among panelists on every item included in the questionnaires. In the first round, several open-ended questions were proposed, mixed with multiple-choice questions and a few statements. In the second round, a majority of multiple-choice questions and statements were used. The latter allowed the panelists to indicate their level of agreement using a 4-point Likert scale: (1) “fully disagree”; (2) “partially disagree”; (3) “partially agree”; and (4) “fully agree”. For analysis, responses showing “full” or “partial” agreement were grouped together, as were those showing “full” or “partial” disagreement, in order to assess how much consensus emerged among binary answers. All responses and free-text comments collected during the first round were reviewed by the Executive Committee. Items that did not reach the prespecified consensus threshold were assessed to identify potential ambiguity or aspects requiring further specification. As appropriate, these items were reworded, divided into more specific questions, or converted into closed questions or statements, while preserving the original construct. New questions were introduced when clinically relevant issues emerged from the round 1 responses. Before voting on a reformulated item, panelists were shown the anonymized aggregate results from the previous round, generally through summary graphs and/or frequency distributions. After reviewing the findings of the first round regarding the most frequent types of adverse events associated with the use of BsAbs, a detailed section on the management of each common type of adverse event was included in the second round.

Survey responses of both Delphi rounds were analyzed in aggregate to protect voter anonymity. Given the relatively small size of the panel and the homogeneity of the clinical specialization of the experts (i.e., all hematologists), subgroup analyses were not performed.

In the Delphi rounds, consensus was determined by calculating the percentage of agreement (for qualitative responses, multiple-choice questions, and statements) or, less frequently, the median value (for quantitative responses). The convergence criterion for consensus was prespecified before the Delphi rounds and was not selected on the basis of the observed results [19,22,23]. In this manuscript, “majority consensus” refers to a consensus achieved by 66.7% to 99.9% of the panelists, whereas “full consensus” denotes unanimous agreement among all panelists. In a rapidly evolving clinical setting, the 66.7% threshold was considered appropriate to identify meaningful agreement while retaining sensitivity to potentially relevant differences in expert opinions.

### 2.4. Panel of Experts

Panelists were hematologists working in Italian referral centers with recognized expertise in MM therapy. A total of 15 hematologists were invited to participate, and efforts were made to involve clinicians from centers in Northern, Central, and Southern Italy.

All the hematologists satisfied the inclusion criteria and completed both rounds of the survey. The study was conducted using SurveyMonkey^®^, an ad hoc online platform for implementing surveys.

### 2.5. Ethics

This work is based on a survey of hematologists who contributed with their expertise and beliefs. No patients were involved in the Delphi process, and the study does not fall within the scope of those requiring ethical approval in Italy (Determine AIFA: 8 August 2024, https://www.gazzettaufficiale.it/eli/id/2024/08/20/24A04320/sg (accessed on 24 June 2026); 20 March 2008, https://www.gazzettaufficiale.it/eli/id/2008/03/31/08A02109/sg (accessed on 24 June 2026)). All clinicians were informed by electronic means of the objectives of the project and study funding before initiating the survey, both in a contact e-mail and in an introductory page of the web survey. All panelists provided their consent to participate in the Delphi study by answering a specific question on the same introductory page of the questionnaire. The study is sponsored by Pfizer.

## 3. Results

### 3.1. Choice of the Optimal T-Cell-Redirecting Therapy in TCE RRMM

In the first section of the questionnaire, the panelists were asked to address two main questions that come up when the multi-phase T-cell-engaging therapy patient journey starts. These included the choice of the initial T-cell immunotherapy (i.e., BsAbs versus CAR-T cells) to be used for the care of patients with TCE RRMM and of the target antigen (i.e., BCMA versus GPRC5D) against which BsAbs are redirected. Clinical decision making regarding the cellular (i.e., CAR-T) vs. non-cellular (i.e., BsAbs) type of immunotherapy is driven by many variables, including availability and logistics, physician- and patient-related factors, disease-related characteristics, prior therapy exposure, and the impact of treatment choice on subsequent treatments. Taking into account all the variables potentially influencing the ultimate choice and assuming equal access to both CAR-T-cell therapy and BsAbs, the panel agreed that the available evidence strongly supports first pursuing CAR-T-cell therapy (Table 1). For those patients who are deemed unsuitable to receive CAR-T cells, a summary of considerations supporting the choice of BsAbs is reported in Table 1.

Additional indications can be found in the Supplementary Materials. Consensus emerged for prioritizing the use of BsAbs over CAR-T in patients with one or more of the following characteristics: frail status according to IMWG (100% agreement—full consensus); patient preference (93% agreement—majority consensus), related to the shorter hospitalization period and the reduced risk of severe CRS and ICANS; the presence of severe cardiovascular disease(s) (87% agreement—majority consensus); advanced age (>80 years; 80% agreement—majority consensus); high ECOG performance status (80% agreement—majority consensus); the presence of severe renal dysfunction (67% agreement—majority consensus); and the presence of severe pulmonary disorders (67% agreement—majority consensus). Regarding the disease characteristics, BsAbs were unanimously preferred over CAR-T-cell therapy in the case of rapidly progressive MM (100% agreement—full consensus), a consensus driven by the immediate, “off-the-shelf” availability of BsAbs.

Given the current lack of anti-GPRC5D CAR-T-cell therapy approved by regulatory agencies for TCE RRMM, the second set of questions was restricted to the choice of the target antigen (i.e., BCMA or GPRC5D) against which BsAbs should be redirected. An anti-GPRC5D BsAb was suggested by 93% of panelists as the first option for patients at high infectious risk (majority consensus), as defined by a prior history of multiple or severe infections, or with severe pulmonary disorders. Notably, when discussing with the patient the pros and cons of GPRC5D as a potential target antigen, the lower risk of infections should be counterbalanced by presenting the possible on-target, off-tumor treatment-related adverse events, because they may adversely impact patients’ quality of life. Conversely, an anti-BCMA BsAb was the preferred first-choice immunotherapy (73% agreement—majority consensus) for patients without a high risk of infections.

### 3.2. Workup Assessments Before Starting T-Cell-Engaging Anti-BCMA BsAbs

In the first Delphi round, the expert panel voted for a list of required clinical and laboratory assessments to be performed before starting therapy with anti-BCMA BsAbs in TCE RRMM patients (Table 2).

An agreement was achieved on the following exams/evaluations: complete blood count (100% agreement—full consensus); renal function (100%—full consensus); liver function (100%—full consensus); ECOG performance status (100%—full consensus); immunoglobulin dosage (100%—full consensus); assessment of HCV (93%—majority consensus); HIV (93%—majority consensus) and HBV (87%—majority consensus) serological status; cardiac function, LVEF (87%—majority consensus); up-to-date vaccinations for pneumococcal disease (87%—majority consensus), seasonal influenza (80%—majority consensus), Varicella-Zoster Virus (80%—majority consensus), and SARS-CoV-2 19 (73%—majority consensus); frailty IMWG score (67%—majority consensus); and assessment of CMV PCR (67%—majority consensus). The key considerations for the assessment of patient eligibility to receive anti-BCMA BsAbs are summarized in Table 2, and additional findings are reported in the Supplementary Materials.

### 3.3. Inpatient Administration of BsAb SUD

Although the EMA labeling guidance for approved BsAbs does not mandate patient hospitalization throughout their SUD, this treatment phase is usually given in an inpatient setting, which allows the close monitoring and prompt treatment, if required, of early symptoms and signs of cytokine release syndrome (CRS) and immune effector cell-associated neurotoxicity syndrome (ICANS). According to the panel, key components of the roadmap for the safe management of BsAb SUD in a hospital setting include the following (Supplementary Table S1): (1) the availability of specifically trained hematology healthcare professionals; (2) an intensive care unit; (3) a highly specialized multidisciplinary team; (4) a detailed protocol summarizing the procedures for the close monitoring of the patient and prompt intervention, if requested, as well as roles and responsibilities; (5) tocilizumab availability.

### 3.4. Outpatient SUD Administration

Five out of fifteen (33%) panelists had ever managed the SUD phase of anti-BCMA BsAbs in an outpatient setting. Nevertheless, all the panelists supported the possible outpatient administration of SUD, provided that selected criteria were met. In particular, the key factors for the appropriate implementation of the outpatient SUD phase were the following (Table 3): (1) 24/7 availability of a reliable caregiver (100% agreement—full consensus); (2) patient residence close to the reference hospital (100% agreement—full consensus); (3) training of both the healthcare personnel (93% agreement—majority consensus) and the patients/caregiver to correctly monitor neurotoxicity and vital signs (87% agreement—majority consensus); (4) 24/7 on-call availability of healthcare personnel (87% agreement—majority consensus); (5) clear definition of the criteria requiring hospitalization (87% agreement—majority consensus); (6) 24/7 availability of a hospital bed, should it become necessary (67% agreement—majority consensus).

In addition, panelists agreed on the need to carefully select patients to whom to offer safe SUD administration in an outpatient setting. The key criteria selected by the panel were patient cognitive status (100% agreement—full consensus), patient/caregiver education on how to promptly recognize treatment-related adverse events (100%—full consensus), ECOG performance status score <3/absence of comorbidities that, if present, should be well controlled (93%—majority consensus), absence of high tumor burden (80%—majority consensus), or quickly progressing disease (67%—majority consensus).

### 3.5. Patient Monitoring

According to the panelists, a complete baseline laboratory evaluation including complete blood counts, and liver and renal function is required (93% agreement—majority consensus). A C-reactive protein, procalcitonin, ferritin, fibrinogen, and coagulation panel and LDH test should be performed in all patients before the start of BsAb therapy. Baseline neurological evaluation and careful patient monitoring for signs of CRS and immune effector cell-associated encephalopathy (ICE) scores are recommended after each step-up dose and the first full dose. Recommendations on patient monitoring during anti-BCMA BsAb treatment, as emerged from the panel, are summarized in Table 4.

Full agreement was found in the second round (after an exploratory, open-ended question in the first round) that the main clinical parameters to be monitored during SUD are blood pressure, body temperature, respiratory parameters, and ICE score (100% agreement).

### 3.6. Prevention and Management of Treatment-Related Adverse Events

During the first round, panelists were asked, through a multiple-choice question, to identify the most frequent adverse events associated with anti-BCMA BsAb treatment in patients with TCE RRMM. The key adverse events identified were infections, CRS, hematological toxicities/cytopenia, ICANS, and other neurological toxicities (93% agreement—majority consensus). During round 2, prevention and management strategies were investigated separately for each adverse event. The main findings are summarized in Supplementary Tables S2 and S3, while a thorough description of additional findings is provided in the Supplementary Materials.

**Infections**: Regarding the risk of infections, which are predominantly bacterial and viral, but also opportunistic (more commonly including *Pneumocystis jiroveci* pneumonia and CMV reactivation), all panelists (100%—full consensus) agreed that they are a relevant burden for patients receiving BsAbs. The risk of grade ≥ 3 infectious episodes is even higher when the target antigen is BCMA compared to GPRC5D. A high convergence emerged on several key strategies for preventing and managing infections in TCE RRMM patients treated with anti-BCMA BsAbs. Regarding the timing of onset, 60% of panelists reported that infections occur more frequently in the initial phase (<100 days), though the risk remains high even in the long term. This finding led 33% of experts to perceive the risk as stable over time. A high convergence between experts was found on the key strategies for preventing, mitigating, and managing infections in patients treated with BsAbs. In particular, there was a high level of agreement on prophylactic strategies beyond immunoglobulin replacement therapy. Overall, 93% of panelists supported antiviral and anti-*Pneumocystis jirovecii* prophylaxis with both acyclovir and trimethoprim–cotrimoxazole, while for 79%, vaccination strategies were a key factor (majority consensus). The vaccines most frequently used by panelists in their real-world clinical practice included seasonal influenza, SARS-CoV-2, pneumococcal, and varicella-zoster virus, particularly the recombinant adjuvanted herpes zoster vaccine. Vaccinations against encapsulated bacteria (e.g., Haemophilus influenzae type B and meningococcal serogroups A, B, C, W, and Y) and respiratory syncytial virus were also reported in selected responses. Prophylactic immunoglobulin administration was supported by 80% of participants (majority consensus). Among these, a pre-emptive approach based on an IgG threshold of 400 mg/dL was reported by 50%, while 33% supported a primary prophylactic approach regardless of IgG concentration and 17% indicated other strategies. All panelists indicated a dosing frequency of every four weeks (100%—full consensus), and immunoglobulins were administered intravenously in 67% of cases (majority consensus). Prophylaxis was maintained for the entire duration of treatment in 67% of responses (majority consensus). Finally, 83% of experts agreed that training healthcare personnel, patients, and caregivers is necessary to improve the recognition and management of infections during anti-BCMA BsAb treatment (majority consensus).

With reference to the treatment of infectious adverse events, experts reached full consensus on the necessity of broad-spectrum or antibiogram-targeted antibiotic therapy. Ninety-three percent of panelists also recommended a targeted antiviral therapy when an infection is documented (majority consensus).

**CRS**: Consensus was reached on the timing of CRS onset, with almost all experts reporting step-up doses and the first full dose as the most critical phases (93% agreement—majority consensus). Experts agreed on the key symptoms and signs of CRS, which range from isolated fever (full consensus) to, if not promptly treated, hypotension (87% agreement—majority consensus), hypoxia, and dyspnea (these latter two with a 60% agreement, missing the 67% threshold). According to most panelists, the diagnostic workup of CRS is primarily aimed at excluding infections, due to overlapping symptoms and signs, and includes a physical examination, inflammatory markers, blood cultures, and a chest X-ray (92% of experts—majority consensus).

The management strategies for CRS reached high convergence on the use of tocilizumab as early as possible and even if grade 1 (e.g., isolated fever > 38 °C) persists for >24 h (93% agreement—majority consensus). Steroids were recommended as a second-line therapy if CRS persists or recurs after tocilizumab use (87% agreement—majority consensus).

**Hematological toxicities/cytopenia**: No agreement was reached among experts on the timing of the onset of treatment-related hematological toxicities/cytopenia. For 47% of them, the highest peak occurred earlier in the treatment phase (≤30 days) and was possibly mediated, at least in part, by the cytokine storm associated with CRS, while for an additional 47% of experts, hematopoietic recovery might occur later on (>30 days). Conversely, there was full consensus on the use of the granulocyte colony-stimulating factor (G-CSF) growth factor to treat grade ≥3 neutropenia.

**ICANS**: ICANS is one of the three most frequent neurological adverse events reported with BsAb therapy, the remaining two being headache and peripheral neuropathy. A high level of consensus was reached on all questions regarding the occurrence and management of ICANS. A majority consensus was reached that ICANS occurs most frequently during the SUD phase or within cycle 1 (92% agreement). The most reported symptoms and signs ranged from confusion (80% agreement) and aphasia or dysgraphia (67% agreement) to severe encephalopathy. According to the experts, the diagnostic workup of ICANS should include an MRI or CT scan (87% agreement) in patients with grade ≥2 neurological toxicity, who should also undergo an EEG, together with close neurological monitoring based on the ICE score (80% agreement).

There was full consensus on the standard use of dexamethasone to treat grade ≥2 ICANS or high-dose prednisolone in patients refractory to dexamethasone, and the involvement of a multidisciplinary team (including a neurologist) to manage these types of adverse events.

**Non-ICANS neurological toxicities**: Unlike CRS, ICANS, and headaches, which were reported to typically occur during the early treatment phases, most of the experts (73%—majority consensus) agreed that several infrequent non-ICANS neurological complications, such as cranial nerve palsy and sensory peripheral neuropathy, might also occur in later treatment phases.

Multiple key signs and symptoms were reported to aid the clinical identification of these adverse events, but none met the consensus threshold. For the diagnostic workup, a majority consensus was reached on the use of the following procedures/tools: (1) a specialist neurological examination (73% agreement); (2) imaging, i.e., MRI and/or CT scans (67% agreement); (3) electromyography (67% agreement).

The management of other neurological toxicities was considered strictly multidisciplinary, with the neurologist identified as the primary specialist to be involved (100% agreement—full consensus). Most hematologists agreed that treatment decisions should be shared with a specialized neurologist and depend on the type of neurological complication (specific treatments such as pregabalin and steroids were mentioned by 33% of hematologists). Further, long-term patient monitoring should be conducted through periodic visits and in close coordination with a neurologist (80% agreement—majority consensus).

### 3.7. BsAb Dosing Schedule

The approved anti-BCMA T-cell-engaging BsAbs teclistamab and elranatamab are administered with a weekly schedule over the first 6 months; a reduced dosing frequency every 2 weeks is allowed after 6 months for elranatamab in patients achieving any type of response, or in the case of teclistamab, in those achieving a complete response sustained for at least 6 months [24,25]. Moreover, a 4-week schedule is now approved for elranatamab after 1 year of treatment. In line with these indications, 100% of panelists considered a less dose-dense treatment schedule appropriate in patients achieving a (deep) response to mitigate the risk of infections.

### 3.8. Anti-BCMA BsAbs in Special Patient Populations

The level of consensus on the feasibility of anti-BCMA BsAb treatment across several challenging patient subgroups was generally high (Table 5). During the first round, majority consensus was reached on the feasibility of anti-BCMA BsAb treatment in the following subgroups: (1) frail patients, whether frailty was defined according to the International Myeloma Working Group (IMWG)/IMF score (93% agreement), advanced age (92% agreement), or the presence of comorbidities (85% agreement, depending on the specific type of underlying condition); (2) patients with high-risk cytogenetics (93% agreement—majority consensus); (3) patients with extramedullary disease (93% agreement—majority consensus); (4) patients with ESRD (86% agreement); (5) patients with active PCL (79% agreement); (6) patients with CNS involvement (67% agreement).

During the second round, we asked a group of questions to evaluate the feasibility of CAR-T-cell therapy as opposed to anti-BCMA BsAbs in the same selected subgroups. Advanced age (>80 years) was considered compatible with CAR-T treatment by 69% of the experts (majority consensus), a finding discordant with the 67% agreement on non-feasibility of CAR-T-cell therapy in frail patients according to the IMWG/IMF score. CAR-T-cell therapy was also considered non-feasible in patients with ESRD (67% agreement—majority consensus), defined as those requiring dialysis/transplant or with eGFR < 15 mL/min per 1.73 m^2^ for at least 4 weeks.

### 3.9. Optimal Sequencing of T-Cell Redirecting Immunotherapies

Regarding the optimal sequencing of T-cell-redirecting therapies, the experts recognized that it actually remains an area of active research. Among them, 93% agreed on the possible use of anti-BCMA BsAbs after prior CAR-T-cell therapy targeting the same antigen, an option further supported if the treatment-free interval is of at least 9 months (Table 6). If relapse after anti-BCMA CAR-T cells occurs earlier, experts suggested switching the target antigen and offering a GPRC5D-targeting BsAb. This latter option was strongly recommended also in those patients for whom a sequential use of BsAbs targeting BCMA at first, and GPRC5D later on, may be advisable.

The panel agreed on the use of an anti-GPRC5D BsAb as a bridging therapy to CAR-T (voted by 79% of panelists; although the target was not specifically investigated, 3 out of 11 panelists specified in an open field to use a BsAbs targeting GPRC5D in this case). Consensus was reached that, at least hypothetically (as the scenario is not currently permitted in Italy), CAR-T therapy could be a beneficial option for patients relapsing after anti-BCMA BsAb treatment, after a therapy-free interval of at least 6–12 months (73% agreement).

## 4. Discussion

BsAbs targeting BCMA and GPRC5D were primarily administered in academic hospitals. More recently, their use has also been expanded in community, non-academic centers, thus offering remarkable therapeutic advancements for a growing number of difficult-to-treat patients, but also raising clinical challenges. In fact, these agents are relatively new in the treatment landscape for TCE RRMM, and many healthcare professionals may have limited familiarity with their optimal and safe management.

The successful transition of bispecific T-cell-engaging antibodies from highly specialized hospitals into local health centers requires the adequate education of healthcare professionals and thoughtful care coordination between practice centers and referral hospitals. By establishing network partnerships with referral hospitals, local centers may offer their patients innovative treatment options and narrow potential treatment disparities. Addressing the gaps existing in centers lacking an established BsAb treatment program (including a well-trained multidisciplinary team, site-specific standard operating procedures, protocols guiding the entire treatment program, identification of roles and responsibilities, patient selection criteria, SUD procedure, patient and caregiver education) enables safe and effective BsAb administration, thus ensuring that more TCE RRMM patients have access to community onco-hematology centers. This manuscript aims at optimizing patient care at each phase of their journey and improving the healthcare profession education process by providing practical considerations from experienced hematologists in BsAb-based therapy for TCE RRMM patients. For this purpose, expert advice on key issues surrounding the roadmap for T-cell redirecting therapies, with particular emphasis on BsAbs, were collected using a modified Delphi consensus methodology. Some of the recommendations herein provided are based on the authors’ experience in clinical practice, rather than on robust evidence from clinical studies and should be considered as expert opinions complementary to current guidelines. Several clinical challenges related to the Italian healthcare system were also addressed, thus ensuring tailored country-specific recommendations and a customized guidance for the management of TCE RRMM according to the national regulatory landscape in Italy.

### 4.1. Choice of Therapy and Optimal Sequencing of T-Cell-Redirecting Treatments

The panel recognized that many factors, including access and availability, product safety and efficacy profiles, patient fitness and personal preference, disease-related characteristics, physician experience, and treatment sequencing, guide the choice of T-cell-redirecting immunotherapy to be offered to a patient with TCE RRMM. In addition, the lack of head-to-head comparisons between different T-cell-redirecting treatment modalities and uncertainties regarding the best sequencing make the choice increasingly complex [9]. Based on available evidence, and assuming equal access to and eligibility for both CAR-T-cell therapy and BsAbs, the experts suggested pursuing CAR-T-cell therapy first given the higher response rate, durable remission, and quality-of-life benefits from treatment-free intervals that the one-shot CAR-T-cell infusion can provide compared to BsAbs. In addition, results from several studies suggest that efficacy outcomes with BsAbs given as salvage therapy after prior CAR-T-cell infusion are likely to be better than vice versa [26,27]. However, the strict eligibility criteria for receiving CAR-T and logistical and/or manufacturing bottlenecks make BsAbs a more accessible and readily available treatment option for a wider patient population [28]. With cilta-cel now approved in Italy in the early-relapse setting, the sequencing of anti-BCMA CAR-T therapy and subsequent anti-BCMA BsAbs, following an adequate treatment-free interval, is likely to become an increasingly relevant clinical scenario in the future.

Consistent with these considerations, experts agreed on several mixed factors associated with a preference for the use of BsAbs over CAR-T, ranging from patient features (i.e., indicators of frailty, including elderly age, presence of severe comorbidities, compromised renal function, and ECOG performance status) and patient preferences (i.e., reduced hospitalization time [29]) to MM characteristics (i.e., aggressive/rapid disease, extramedullary disease) and treatment-related features (i.e., treatment history with a focus on earlier target therapies, sequencing, and accessibility off-the-shelf). Discrepancies found regarding the possible use of CAR-T-cell therapy in older patients and its preferential avoidance in frail subjects [30] may simply reflect the separate framing of the questions and underscore the need to individually assess CAR-T-cell eligibility in patients with advanced age and possible coexistence of comorbidities. CAR-T-cell therapy was also considered non-feasible in patients with ESRD, for whom anti-BCMA BsAbs were preferred. With regard to disease-related characteristics, the panel agreed on the possible use of BsAbs in selected clinical settings (Table 1), as in the case of rapidly progressive disease, primarily driven by their immediate availability and feasibility in patients requiring prompt treatment. However, these findings should be interpreted with caution. Available evidence from pivotal trials and real-world studies consistently support the efficacy of CAR-T-cell therapies in patients with extramedullary disease [31,32]. In clinical practice, treatment selection between CAR-T-cell therapies and BsAbs should therefore be individualized, taking into account logistical and manufacturing factors, disease- and patient-related characteristics, and patient and physician preferences. Emerging strategies, including dual-targeting (e.g., anti-BCMA and GPRC5D) BsAbs, may help to partially address this unmet need [33]; however, the current evidence remains limited and further investigation is warranted.

Similarly, the panel consensus indicating the limited feasibility of CAR-T-cell therapy in patients with end-stage renal disease should be contextualized. Recent real-world studies have demonstrated that CAR-T-cell therapy can be administered safely and effectively in patients with severe renal impairment [34], including those requiring dialysis, albeit with a longer duration of cytopenias compared with patients without renal dysfunction.

In this setting, both CAR-T-cell therapy and bispecific antibodies should be considered potentially feasible options, with the treatment choice guided by overall patient fitness, expected hematologic recovery, infectious risk, and local expertise. The more prolonged cytopenias observed after CAR-T-cell therapy may, in some cases, favor the use of bispecific antibodies; however, this should not be interpreted as a contraindication to CAR-T-cell therapy per se.

In recent real-world studies, the effectiveness and safety of anti-BCMA BsAbs in several challenging patient populations were confirmed, although sometimes with decreased response rates and progression-free survival as compared to overall TCE RRMM patients [16,35,36].

Overall, our findings do not aim to redefine therapeutic hierarchies between CAR-T-cell therapies and bispecific antibodies in these cases, but rather to provide a pragmatic framework to support individualized treatment decisions in real-world clinical practice. By integrating expert consensus with available evidence, and by focusing on the specific constraints of the Italian healthcare system, the present expert analysis highlights the clinical scenarios in which bispecific antibodies may represent a valuable and feasible therapeutic option, particularly when access to cellular therapies is limited or delayed.

Regarding the sequencing of treatment lines across disease relapses, a switch of the target antigen and the type of T-cell-redirecting immunotherapy (i.e., cellular vs. non-cellular) at the time of relapse after prior immunotherapy should be the primary treatment option. Currently available data suggest that a switch from a BCMA- to GPRC5D-targeted immunotherapy is more effective than the sequential use of different BCMA-directed modalities. Similarly, a switch from GPRC5D-targeted therapy to BCMA-directed treatment can be an effective option for the treatment of relapsed/refractory MM. If switching the target antigen after previous BCMA exposure is not possible, then a second, different type of BCMA-directed immunotherapy can be considered, particularly if CAR-T-cell therapy is followed by BsAbs. Consistent with this sequencing, experts agreed that patients progressing after anti-BCMA CAR-T-cell therapy may still benefit from a BsAb targeting the same antigen, particularly if the treatment-free period is at least 6 to 9 months. This is consistent with the results of clinical trials and real-world studies, showing reduced but still satisfactory clinical benefits and/or response rates to anti-BCMA treatments (including both CAR-T and BsAbs) in subgroups with prior BCMA-directed therapy exposure [26,27,35,36,37]. Despite current Italian regulatory indications permitting bispecific antibody use after CAR-T therapy but not vice versa, most experts agreed that CAR-T therapy, when feasible, could reasonably be offered to patients relapsing after anti-BCMA bispecific antibody treatment. Available data from the literature are very limited [37,38], and evidence on such a type of treatment sequencing is currently weak. In addition, performing tests to exclude BCMA loss and biallelic BCMA deletion as well as point mutations or in-frame deletions in the BCMA gene would be extremely informative when sequential anti-BCMA immunotherapies are planned.

### 4.2. Pre-Treatment and On-Treatment Management

The workup before starting any T-cell-redirecting therapy is critical for selecting the most appropriate treatment and for identifying the optimal target antigen, as well as predicting the risk of possible treatment-related AEs and promptly starting adequate prophylactic measures. This roadmap involves a comprehensive assessment of disease status, organ function, infection risk, and logistical planning of step-up doses to be delivered in either an inpatient or outpatient setting. The panelists expressed their agreement on pre-treatment assessments, providing a better definition of the relevance of different variables to assess among those recommended by the summary of product characteristics. According to the expert opinion, the main clinical parameters to be monitored during SUD are blood pressure, body temperature, respiratory parameters, and immune effector cell-associated encephalopathy (ICE) scores.

Only one-third of the panelists had direct experience in the outpatient management of the SUD phase of anti-BCMA BsAbs, but all other participating experts expressed a favorable opinion towards this option. Recent guidelines and consensus documents discussed the possibility of managing BsAb SUD in an outpatient setting. The IMWG Guidelines recommended developing operating protocols to optimize the local care management of BsAb treatment [13]. In the UK consensus for the effective introduction of anti-BCMA BsAbs for RRMM, 90% of experts tended to agree or strongly agreed that, as the clinical experience with BCMA BsAbs grows, low-risk patients may be able to undergo SUD in an ambulatory care setting, providing that suitable protocols are in place for the monitoring and escalation of care if required [39]. Early real-world data from a US study of 58 patients with MM treated with teclistamab, under an outpatient model for SUD, showed that the scheme was feasible and safe, and was associated with improved patient experience and reduced healthcare resource use [40]. Thus, increasing evidence supports the potential conduct of anti-BCMA BsAb SUD in an ambulatory care setting, provided that prophylaxis strategies are met and suitable conditions and procedures are available. The first Italian real-world experience with outpatient SUD of bispecific antibodies (elranatamab, teclistamab, talquetamab), presented at the latest SIE Congress, provides early and practice-informing evidence supporting the feasibility of this approach [41]. Data from the Bologna center indicate that outpatient SUD can be safely implemented in selected patients, without an increased incidence or severity of immune-mediated toxicities compared with inpatient administration. In the outpatient cohort, CRS events were limited to grade 1 and did not require hospitalization. In addition to label-recommended premedication, most patients received dexamethasone at 20 mg as a preventive strategy, administered after each step-up dose or after the completion of SUD and the first full dose, with minimal use of tocilizumab.

Importantly, the feasibility of outpatient SUD should not be interpreted as a simplification of care, but rather as a structured and highly regulated process. Appropriate patient selection remains crucial and includes the careful evaluation of performance status, disease aggressiveness, comorbidities, cognitive function, and social support. The education of both patients and caregivers, the availability of a trained multidisciplinary team, and clear protocols for the escalation of care are mandatory prerequisites.

Differences across healthcare systems should be acknowledged. Although the prophylactic use of tocilizumab is widespread in many countries, in Italy, its availability in this setting is quite heterogeneous. When unavailable, dexamethasone-based preventive strategies may be considered while carefully assessing infection risk.

### 4.3. Adverse Events and Treatment Schedule

Patients treated with anti-BCMA BsAbs have an increased occurrence of infectious adverse events, mainly associated with treatment-induced immunosuppression [42]. In this context, immunoglobulin replacement therapy should be regarded as an essential component of supportive care in patients treated with anti-BCMA BsAbs, given the depth and persistence of treatment-related humoral immunosuppression. Two main strategies are currently adopted in clinical practice: a primary prophylactic approach, as recommended by the IMWG, and a pre-emptive, IgG-guided replacement strategy [13]. Notably, emerging literature published after the most recent IMWG guidelines supports the prophylactic use of immunoglobulins in patients receiving BsAbs, showing that prophylaxis should be initiated regardless of baseline IgG levels, and providing a strong rationale for a broader and earlier implementation of this strategy to mitigate infectious risk [43].

While the optimal approach remains debated, both were considered appropriate by the expert panel, with increasing real-world experience suggesting that systematic pre-emptive immunoglobulin supplementation throughout treatment may be particularly beneficial in patients receiving continuous BCMA-directed therapy.

De-escalation from weekly to biweekly or monthly dosing was shown to decrease the incidence of severe infections without compromising efficacy outcomes in the long term [44,45]. Notably, a decrease in the new onset of severe infections was reported with a less intensive dosing schedule adopted after the first 6 months of therapy. Moreover, safety and efficacy data were reported for elranatamab after switching the treatment schedule to the 4-week approved administration, showing that reducing the dosing frequency may improve safety without compromising efficacy: following the switch from Q2W to Q4W dosing, 92.6% of patients maintained their responses ≥6 months after the switch, and among patients who switched to Q4W dosing, the incidence of grade 3/4 infections decreased from 17.9% to 10.7% [45]. Consistently with these data, a key recommendation emerging (with full consensus) from our study is the need, after the anti-BCMA BsAb response, to implement adequate treatment de-escalation strategies to decrease the risk of toxicity.

Treatment de-escalation, including reducing the dosing frequency to Q2W or Q4W after an initial response, represents a clinically relevant option in anti-BCMA bispecific antibody therapy for reducing long-term toxicity—particularly infectious risk—while improving treatment tolerability and manageability in routine practice, without compromising efficacy and with a potential beneficial impact on T-cell exhaustion.

However, relevant differences exist among currently available agents. Both teclistamab and elranatamab require an initial period of weekly administration of at least six months. Thereafter, de-intensification may be considered according to treatment-specific criteria: the achievement of any response for elranatamab and sustained CR for teclistamb.

The approval of extended dosing intervals for elranatamab, including monthly administration, provides an additional opportunity to improve long-term tolerability without compromising efficacy, as supported by data showing that, among 27 patients who switched to Q4W dosing at least 6 months before the data cutoff, 92.6% maintained their response for ≥6 months after the switch, including 88.0% who sustained a ≥CR [45].

These differences underline the importance of tailoring de-escalation strategies to the specific BsAb used, rather than adopting a uniform approach.

Although neurotoxicity was not the primary focus of the discussion, its clinical relevance warrants consideration. The panel confirmed that immune effector cell-associated neurotoxicity syndrome and other neurological adverse events are generally manageable with established protocols and do not substantially differ across anti-BCMA bispecific antibodies. Early recognition, standardized monitoring using the ICE score, and prompt multidisciplinary intervention remain essential components of safe clinical practice.

Panelists highlighted no difference in the occurrence of adverse events, or in other clinical aspects, according to the use of distinct, currently available, anti-BCMA BsAbs.

### 4.4. BsAb Feasibility in Special Populations

Finally, the feasibility of bispecific antibody therapy across several challenging patient populations—including frail patients and those with high-risk cytogenetics, extramedullary disease, central nervous system involvement, and end-stage renal disease—emerged as a distinctive strength of this therapeutic approach. While outcomes may be inferior in some subgroups compared with the overall TCE RRMM population, the ability to offer an effective, off-the-shelf immunotherapy in these settings represents a meaningful advance in real-world care.

In this context, a key objective of the present work is to provide practical guidance on the use of anti-BCMA BsAbs particularly in less consolidated clinical scenarios where evidence and consensus remain limited. After the publication of the most recent IMWG recommendations [13], some data from real-world experiences emerged for these patients excluded by clinical trials, allowing for a more nuanced interpretation of optimal management strategies in these subgroups, in agreement with the expert recommendations that emerged in the present work. For instance, early signals of activity have begun to emerge in particularly high-risk and understudied settings such as CNS involvement and plasma cell leukemia [46,47]. Moreover, real-world and clinical experience suggests that BsAb can be safely administered in patients with severe renal impairment, including those requiring dialysis without the need for dose modifications [48,49]. Although evidence remains limited and caution is warranted in these subgroups, these observations highlight a potential new therapeutic frontier in which T-cell engagers may expand treatment opportunities for patient populations historically characterized by extremely poor outcomes.

### 4.5. Study Limitations

Among the main study limitations are those commonly associated with consensus methodologies [50]. Although we adopted a rigorous, structured Delphi process, the level of evidence associated with the collection of opinions from expert panels is low. Therefore, the related recommendations should be interpreted as expert opinion rather than definitive evidence-based guidance. Nevertheless, in an area where robust quantitative evidence remains scarce, expert consensus may help address important knowledge gaps and inform clinical practice while awaiting validation through larger prospective studies and real-world evidence. An additional limitation is that ide-cel and cilta-cel were not assessed separately, reflecting the limited Italian clinical experience with cilta-cel at the time of the survey. Therefore, CAR-T-cell findings should be interpreted at the class level and may not capture product-specific differences. All invited clinicians were from Italy, and the generalizability of our findings to other countries may be limited. However, most topics considered in the consensus were not peculiar to a specific national setting but were rather of general interest to clinicians worldwide. Furthermore, some specific topics investigated may present important practical differences across countries (e.g., the occurrence of adverse events and, specifically of infection types, may vary in different geographic areas), and thus, the availability of country-specific opinions is important. Information bias may also be an issue, given the high number of topics explored and thus the relatively long duration of the survey. The strengths of the study include the following: (1) compliance with Delphi methodology requirements and with ACCORD reporting guidelines [20]; (2) the full participation of the panelists invited to both Delphi rounds; (3) the inclusion of a homogeneous group of 15 hematologists who met a priori defined criteria; (4) the involvement of expert clinicians practicing at referral centers for the disease, thus providing recommendations useful to Italian physicians treating MM in less specialized settings; (5) the low frequency of missing responses to each single question, as no item of the questionnaire required additional investigation due to >33% of non-responders.

## 5. Conclusions

The rapid integration of anti-BCMA bispecific antibodies into the treatment landscape for multiple myeloma has created an urgent need for practical guidance to support their safe and effective use in real-world settings. This Italian modified Delphi consensus addresses this gap by providing a structured, expert-driven clinical guide that accompanies clinicians through all critical phases of BsAb therapy in triple-class-exposed relapsed/refractory multiple myeloma.

By combining expert consensus with available evidence, this work goes beyond theoretical recommendations and delivers concrete, actionable indications for patient selection, treatment sequencing, monitoring strategies, management of adverse events, and treatment de-escalation, including complex and under-represented patient populations. Generated as a pragmatic contribution to real-world clinical practice, these recommendations should be interpreted as complementing, rather than replacing, current evidence-based guidelines within individualized treatment decision making. The resulting framework is intended to serve as a strategic clinical reference for hematologists at different levels of experience, facilitating the responsible adoption of anti-BCMA BsAbs and contributing to the optimization of patient care in an evolving therapeutic landscape.

## Figures and Tables

**Table 1:** Key considerations for selecting the optimal T-cell-redirecting treatment strategy (CAR-T-cells or BsAbs) and the target antigen (BCMA or GPRC5D) for the management of TCE RRMM.

Considerations	Agreement Level (%)	Degree of Consensus ^1^	Round of Agreement
**1. Prioritization of CAR-T or BsAbs as first option**			
Prioritize CAR-T if available and feasible	73%	Majority consensus	Round 2
Prioritize BsAbs if CAR-T-cell therapy is unavailable or unfeasible	73%	Majority consensus	Round 2
**2. Factors supporting the primary choice of BsAbs**			
**Patient-related**			
Frailty	100%	Full consensus	Round 1
Preference (reduced hospital stay and/or lower risk of severe CRS and ICANS/delayed neurological toxicities)	93%	Majority consensus	Round 1
Severe cardiac comorbidities	87%	Majority consensus	Round 2
Age >80 years	80%	Majority consensus	Round 1
High (≥3) ECOG performance status	80%	Majority consensus	Round 1
Severe pulmonary comorbidities	67%	Majority consensus	Round 2
Severe kidney comorbidities	67%	Majority consensus	Round 2
**Disease-related**			
Rapidly progressive disease	100%	Full consensus	Round 1
**Other**			
Treatment sequencing (after failure of prior anti-BCMA CAR- T-cell therapy)	73%	Majority consensus	Round 1
**3. Factors driving the choice of the antigen targeted by BsAbs**			
**Patient-related**			
GPRC5D in patients with a prior history of multiple or severe infections	93%	Majority consensus	Round 2
BCMA in patients without high risk of infections and not eligible for CAR-T-cell therapy	73%	Majority consensus	Round 2
**Disease-related**			
No relevant factor	80%	Majority consensus	Round 2
**4. Other factors**			
No relevant factor	73%	Majority consensus	Round 1

^1^ Degree of consensus was defined a priori as follows: majority consensus indicated agreement among 66.7–99.9% of panelists, whereas full consensus indicated unanimous agreement (100%).

**Table 2:** Summary of the key clinical and laboratory assessments to be performed before starting anti-BCMA BsAbs in TCE RRMM patients.

Indication	Agreement Level (%)	Degree of Consensus ^1^	Round of Agreement
**1. Clinical and laboratory assessments before starting treatment**			
Complete blood count	100%	Full consensus	Round 1
Renal function	100%	Full consensus	Round 1
Liver function	100%	Full consensus	Round 1
ECOG performance status	100%	Full consensus	Round 1
Immunoglobulin dosage	100%	Full consensus	Round 1
Baseline assessment/confirmation of HCV serological status	93%	Majority consensus	Round 1
Baseline assessment/confirmation of HIV serological status	93%	Majority consensus	Round 1
Baseline assessment/confirmation of HBV serological status	87%	Majority consensus	Round 1
Cardiac function, LVEF	87%	Majority consensus	Round 1
Vaccination status, pneumococcus	87%	Majority consensus	Round 1
Vaccination status, influenza	80%	Majority consensus	Round 1
**Vaccination status, Varicella-Zoster Virus**	80%	Majority consensus	Round 1
Vaccination status, COVID-19	73%	Majority consensus	Round 1
Frailty score, IMWG	67%	Majority consensus	Round 1
Baseline assessment/confirmation of CMV serological status	67%	Majority consensus	Round 1
**2. Required prophylaxis/preventive actions to be performed before starting treatment**			
**Anti-Varicella-Zoster Virus prophylaxis**	100%	Full consensus	Round 1
With acyclovir 400 or 800 mg/d	73%	Majority consensus	Round 1
Anti-Pneumocystis prophylaxis	100%	Full consensus	Round 1
With Bactrim 2 tablets per 2 d/week or 1 per 3 d/week ^2^	67%	Majority consensus	Round 1
Immunoglobulin supplementation	93%	Majority consensus	Round 1
Anti-HBV prophylaxis ^3^	67%	Majority consensus	Round 1
Monthly monitoring of CMV	67%	Majority consensus	Round 1
**3. Different exams/actions to be performed according to different anti-BCMA BsAbs**			
Clinical evaluations do not differ according to distinct anti-BCMA BsAb treatments	100%	Full consensus	Round 1

^1^ Degree of consensus was defined a priori as follows: majority consensus indicated agreement among 66.7–99.9% of panelists, whereas full consensus indicated unanimous agreement (100%). ^2^ In patients intolerant to trimethoprim–sulfamethoxazole, atovaquone or inhaled pentamidine may be considered for prophylaxis. ^3^ Anti-HBV prophylaxis is indicated for seropositive patients at risk of reactivation.

**Table 3:** Key considerations regarding the outpatient management and administration of BsAbs in TCE RRMM patients.

Indication	Agreement Level (%)	Degree of Consensus ^1^	Round of Agreement
**1. Key procedures to be adopted—Outpatient setting**			
24/7 availability of a reliable caregiver	100%	Full consensus	Round 1
Patient residence near to the reference hospital	100%	Full consensus	Round 1
Training of the healthcare personnel on monitoring neurotoxicity and vital signs	93%	Majority consensus	Round 1
Training of the caregiver on monitoring neurotoxicity and vital signs	87%	Majority consensus	Round 1
24/7 on-call availability of healthcare personnel	87%	Majority consensus	Round 1
Clear definition of the criteria requiring hospitalization	87%	Majority consensus	Round 1
24/7 availability of a hospital bed, should it become necessary	67%	Majority consensus	Round 1
**2. Requirements for an outpatient management of step-up dosing—Outpatient setting**			
Patient status	100%	Full consensus	Round 1
Presence of a reliable, trained caregiver	100%	Full consensus	Round 1
Proximity of the patient residence to a hospital center	100%	Full consensus	Round 1
Maximum of 30 min distance	67%	Majority consensus	Round 1
Absence and/or stability of patient comorbidities	93%	Majority consensus	Round 2
Absence of a high burden of disease	80%	Majority consensus	Round 1
Absence of a quickly progressing disease	67%	Majority consensus	Round 1

^1^ Degree of consensus was defined a priori as follows: majority consensus indicated agreement among 66.7–99.9% of panelists, whereas full consensus indicated unanimous agreement (100%).

**Table 4:** Summary of key considerations regarding patient monitoring during anti-BCMA BsAb treatment.

Indication	Agreement Level (%)	Degree of Consensus ^1^	Round of Agreement
**1. Required clinical and laboratory exams to be performed—Inpatient setting ^2^**			
At baseline, blood tests are required in a hospitalized patient for the evaluation of complete blood count, liver function, and renal function	93%	Majority consensus	Round 2
Clinical and laboratory exams ^3^			
Complete blood count	93%	Majority consensus	Round 2
Renal function	93%	Majority consensus	Round 2
Liver function	73%	Majority consensus	Round 2
Clinical and laboratory exams that should be performed if the patient has fever			
PCR analysis	93%	Majority consensus	Round 2
Blood cultures	80%	Majority consensus	Round 2
Complete blood count	73%	Majority consensus	Round 2
**2. Required clinical and laboratory exams to be performed—Outpatient setting**			
At baseline, exams required in an outpatient setting do not differ from those in hospitalized patients	83%	Majority consensus	Round 2
Management in an outpatient setting if the patient has fever			
Symptomatic treatment	67%	Majority consensus	Round 2
**3. Required clinical parameters/factors to be monitored**			
Main clinical parameters/factors to be monitored during step-up dosing are blood pressure, body temperature, respiratory parameters, and immune effector cell-associated encephalopathy (ICE) score	100%	Full consensus	Round 2
**4. Treatment de-escalation ^4^**			
It is appropriate to consider treatment de-escalation in patients achieving response, to reduce the risk of adverse events	100%	Full consensus	Round 2
**5. Any differences in patient monitoring according to distinct anti-BCMA BsAb therapies**			
Patient monitoring does not differ according to distinct anti-BCMA BsAb treatments	93%	Majority consensus	Round 1

^1^ Degree of consensus was defined a priori as follows: majority consensus indicated agreement among 66.7–99.9% of panelists, whereas full consensus indicated unanimous agreement (100%). ^2^ Frequently reported exams to be performed at baseline in a hospitalized patient that did not achieve consensus included viral serologic testing (reported by 60% of panelists), PCR (53%), and LDH level (47%). ^3^ To be monitored before any subsequent administration of anti-BCMA BsAbs. ^4^ No agreement was achieved on specific aspects related to treatment de-escalation such as the type of response (i.e., 60% of experts consider de-escalation when the patient has a very good partial response, and 40% when the response is complete), time-point for considering de-escalation (i.e., 53% at 6 months, and 33% after two consecutive assessments with achievement of the desired response), and dosing schedule (i.e., 47% reported a biweekly schedule, followed by monthly administration).

**Table 5:** Summary of the key considerations for the use of anti-BCMA BsAbs in special populations of patients with TCE RRMM.

Indication	Agreement Level (%)	Degree of Consensus ^1^	Round of Agreement
**1. It is feasible to treat the following challenging patient populations with anti-BCMA BsAbs**			
Frail patients, according to IMWG/IMF score	93%	Majority consensus	Round 1
Patients with high-risk cytogenetics	93%	Majority consensus	Round 1
Patients with extramedullary disease	93%	Majority consensus	Round 1
Frail patients, according to elderly age	92%	Majority consensus	Round 1
Patients with end-stage renal disease	86%	Majority consensus	Round 1
Frail patients, according to the presence of selected comorbidities	85%	Majority consensus	Round 1
Patients with active plasma cell leukemia (PCL)	79%	Majority consensus	Round 1
Patients with central nervous system (CNS) involvement	67%	Majority consensus	Round 1
**2. Any differences in the treatment choice of distinct anti-BCMA BsAb therapies across these challenging patient populations**			
No definite factor could currently suggest choosing a specific anti-BCMA BsAb	93%	Majority consensus	Round 1

^1^ Degree of consensus was defined a priori as follows: majority consensus indicated agreement among 66.7–99.9% of panelists, whereas full consensus indicated unanimous agreement (100%).

**Table 6:** Summary of the key considerations on optimal sequencing of T-cell redirecting immunotherapies in patients with TCE RRMM.

Indication	Agreement Level (%)	Degree of Consensus ^1^	Round of Agreement
**1. Optimal immunotherapies sequencing**			
BCMA in patients with prior exposure to GPRC5D	100%	Full consensus	Round 1
Eligibility to an anti-BCMA therapy after treatment with another agent targeting BCMA	93%	Majority consensus	Round 1
Consider safety profile (may favor both targets, in turn)	80%	Majority consensus	Round 1
Consider previous toxicities (may favor both targets, in turn)	80%	Majority consensus	Round 1
Use of BsAbs as a bridging therapy to CAR-T	79%	Majority consensus	Round 1
CAR-T is potentially beneficial after anti-BCMA BsAb treatment, after a therapy-free interval >6–12 months	73%	Majority consensus	Round 2
Consider patient preferences (may favor both targets, in turn)	73%	Majority consensus	Round 1

^1^ Degree of consensus was defined a priori as follows: majority consensus indicated agreement among 66.7–99.9% of panelists, whereas full consensus indicated unanimous agreement (100%).

## Data Availability

The original contributions presented in this study are included in the article/Supplementary Materials. The original Italian-language questionnaires are available from the corresponding author upon reasonable request. Further inquiries can be directed to the corresponding author.

## Supplementary Materials

The following supporting information can be downloaded at: https://www.mdpi.com/article/10.3390/cancers18162572/s1, Table S1: Summary of the key considerations on the inpatient management and administration of the step-up dosing phase of anti-BCMA BsAbs in TCE RRMM patients; Table S2: Period of occurrence, prevention, clinical manifestations, and diagnostic workup of treatment-related adverse events associated with anti-BCMA BsAbs in patients with TCE RRMM; Table S3: Treatment, multidisciplinary management, training, and product-related differences for treatment-related adverse events associated with anti-BCMA BsAbs in patients with TCE RRMM.
