# Supplementary material for: Optimizing the Real-World Use of BCMA-Targeted T-Cell-Engaging Therapies in Patients with Triple-Class-Exposed Relapsed/Refractory Multiple Myeloma: An Italian Modified Delphi Consensus
Source: Cancers (Basel). 2026 Aug 10;18(16):2572. doi: 10.3390/cancers18162572 (PMC13510884; doi:10.3390/cancers18162572)
Supplement: Supplementary file 1 [file cancers-18-02572-s001.zip › cancers-4428987-supplementary.pdf]

SUPPLEMENTARY MATERIALS

METHODS – ADDITIONAL DETAILS

Preliminary bibliographic review – Search strings used

Different search strings were used to explore several distinct areas of potential interest, including – among others – step-up dosing procedures, adverse events and their management, patient profiling and use of BsAbs in special patient populations. The bibliographic review was based on search strings composed by a fixed part (i.e., "(multiple myeloma OR plasma cell myeloma) AND (triple-exposed OR triple-class-exposed OR penta-exposed OR fourth-line) AND (bispecific antibodies OR BsAb OR elranatamab OR teclistamab OR bcma)") combined to variable parts focused on, in turn, each topic considered (e.g., for step-up dosing procedures, "(step-up dosing OR dosing management OR treatment initiation)".

Executive Committee – Meetings and contacts

Virtual meetings of the Executive Committee were held on 18 November 2024 (preliminary meeting), 20 January 2025 (study design/planning), 21 May 2025 (kick-off meeting of the Delphi phase) and 9 October 2025 (discussion of results). Frequent email contacts between Executive Committee members occurred, particularly during the phases involving the revision of the questionnaires as well as the discussion of findings of the Delphi iterations.

Criteria for consensus – Focus Level

A Focus Level was applied to each item of the survey, with a corresponding activity, as follows:

Focus level A: high level of agreement between respondents (i.e., ≥66.7%); this level did not require any additional investigation in the subsequent iteration

Focus level B: moderate or high variability of replies between respondents; this level required additional investigation in the subsequent iteration

Focus level C: high (i.e., >33%) proportion of non-responders or "I do not know" answers; this level required additional investigation in the subsequent iteration.

Panel of experts – Eligibility assessment

Panel recruitment followed a two-step process comprising an initial selection phase and a subsequent eligibility assessment. First, the Executive Committee identified a pool of Italian hematologists with recognized expertise in multiple myeloma and BsAb treatment based on objective criteria, including authorship of national and international scientific publications, participation in multicentre real-world studies and clinical trials, direct clinical experience in the management of patients with multiple myeloma, and geographic distribution. Second, eligibility was formally assessed using two criteria prespecified by the Executive Committee before the start of the project. Panelists were required to have treated at least three patients with MM using BsAbs (independent of the clinical setting, i.e., including both RCT and real-life patients) and to have managed BsAb-related adverse events in at least two patients with TCE MM. These pragmatic thresholds were intended to confirm an adequate level of direct and relevant clinical experience in addition to the expertise considered during the initial selection phase.

Panel of experts – Main characteristics

The voting panel of 15 experts was from 8 Italian Regions, including Lombardy (n=4 panellists), Piedmont (n=2) and Veneto (n=1) in northern, Tuscany (n=2), Emilia-Romagna, Marche and Lazio (n=1 each) in central, and Sicily (n=3 panellists) in southern Italy. The median number of patients with MM treated with BsAbs in the panellists' clinical experience was 20 (range 4-80), and all 15 participants reported their experience in managing at least 2 patients with BsAbs treatment-associated adverse events. Most panellists (n=10, 67%) practiced in university hospitals ("Azienda Ospedaliero-Universitaria"), two (13%) in Scientific Institutes/Hospitals of Medical Research ("IRCCS"), two (13%) in local hospitals ("Azienda Ospedaliera"), and one (7%) in a general hospital ("Policlinico"). The hospital departments where the panellists worked managed a median of 400 MM patients, 15 of whom were treated with BsAbs, per year.

Delphi iterations

A contact e-mail to introduce the Delphi study and invite the panellists to participate was sent on 3 July 2025 by C.G., on behalf of the Executive Committee. Thereafter, a second email was sent on 4 July 2025, including a link to the first round of the Delphi survey that was active from 4 to 21 July 2025. Regular reminders were sent during the active phase of the Delphi iterations in order to increase participation rate. The second round was active from 28 October to 6 November 2025.

RESULTS – ADDITIONAL DETAILS

Characteristics associated with the choice of BsAbs treatment – Additional findings

Most experts estimated that, in a hub center, less than 40% of TCE RRMM patients are currently treated with CAR-T therapy (47% of panellist voted for a proportion below 20% of patients and 40% for a proportion of 20% to 40% of patients). In the second iteration we asked the following question for TCE RRMM patients: “At present (13 October 2025), would you prioritize the use of CAR-T therapy over BsAbs?”. Seventy-three percent of panellists replied that they would decide on a patient-by-patient basis, with CAR-T therapy being prioritized when a waiting period and the use of bridging therapy were deemed feasible (73% agreement), and BsAbs being prioritized when MM is aggressive and requires prompt treatment (73% agreement).

Age (93% agreement), ECOG performance status (100% agreement) and disease characteristics (80% agreement) were judged as non-relevant in the choice of the BsAbs target.

In a comparison between different anti-BCMA agents (elranatamab, teclistamab, and linvoseltamab), the expert panel reported no significant differentiating factors to prefer one agent over the others (73% agreement) in triple-class exposed RR MM patients.

Clinical assessment of the patient before starting anti-BCMA BsAbs treatment – Additional findings

We asked for the required prophylaxis/preventive actions to be performed before starting treatment with anti-BCMA BsAbs. Anti-Varicella-Zoster Virus and anti-Pneumocystis prophylaxis had full consensus of experts (100% agreement). Lower agreement values referred to the specific acyclovir (400 or 800 mg/d, 73% agreement) and trimethoprim–sulfamethoxazole (2 tablets per 2 d/week or 1 tablet per 3 d/week, 67% agreement) regimens. For patients intolerant to trimethoprim–sulfamethoxazole, atovaquone or inhaled pentamidine may be considered. Immunoglobulin supplementation (93% agreement), anti-HBV prophylaxis (67% agreement) and monthly monitoring of CMV (67% agreement) also reached majority of consensus among experts. Panelists agreed that antibiotic prophylaxis (93% agreement), antifungal prophylaxis (87% agreement) and monthly monitoring of EBV (73% agreement) are not required before starting treatment with anti-BCMA BsAbs.

Experts unanimously agreed that clinical evaluations to be conducted before starting anti-BCMA BsAbs treatment do not differ according to the specific therapy (i.e., elranatamab, teclistamab, or linvoseltamab) to be adopted.

BsAbs step-up dosing phase – Additional findings

No relevant differences in the clinical management of both inpatient (87% agreement) and outpatient (87% agreement) step-up dosing were reported by the panellists as regards the use of different anti-BCMA BsAbs, i.e., elranatamab, teclistamab and linvoseltamab.

Patient monitoring – Additional findings

Questions on patient monitoring in the outpatient setting were proposed to the 6 experts with direct experience in this field. Exams required at baseline in this setting did not differ from inpatients (83% agreement). For patients with fever, symptomatic treatment was agreed upon by 67% of panellists, while 50% recommended conducting exams for possible infections and using tocilizumab if fever persists over 24 hours or grade >1 CRS is suspected.

Prevention and management of adverse events related to BsAbs treatment – Additional findings

Infections. The majority of experts included chest X-ray or CT scans (93% agreement) and blood cultures (80% agreement) in the diagnostic workup. If the patient has fever, appropriate monitoring requires PCR analysis (93% agreement), blood cultures (80% agreement) and complete blood count (73% agreement). Other frequently reported exams were renal function (47% agreement), procalcitonin (PCT) levels (47% agreement) and liver function (40% agreement) tests. At baseline, clinicians tended to prioritize immunoglobulin levels measurement and HBV and HCV screening (60% agreement for each exam) to prevent infectious adverse events, in the absence of consensus on the topic.

The majority of panellists (80%) reported that their hospital/institution lacked specific educational activities focused on the occurrence of BsAbs-related infections.

Furthermore, panellists unanimously recognized the need to involve a multidisciplinary team to manage the occurrence of infections, specifically an infectious disease specialist (100%).

CRS. Among hospitalized patients, 67% of panelists did not recommend additional CRS prophylaxis beyond label-recommended premedication, whereas 93% supported a prophylactic strategy for outpatients. Prophylactic tocilizumab was supported by 71% of panelists, where permitted by local protocols and access conditions.

Educational needs on CRS were widely acknowledged, with 87% of panelists supporting dedicated training for healthcare staff, patients and caregivers.

The involvement of a multidisciplinary team was deemed essential when CRS occurs in a patient (80% agreement), and participation of intensive care unit specialists for severe cases was required according to 83% of clinicians.

ICANS. Regarding prevention of ICANS, 80% of experts did not recommend any prophylaxis or other preventive actions before starting treatment.

As for other adverse events, most experts agreed on the necessity of dedicated training on ICANS for healthcare providers, patients, and caregivers (93% agreement). Educational training should focus on recognizing both general and specific signs of neurotoxicity, including the calculation of the ICE score.

Other neurological toxicities. The most frequently reported signs to identify these types of adverse events were paresthesia and sensory alterations (50% agreement), followed by a wide range of other signs/symptoms including – among others – headache, tremors, parkinsonism, postural instability, and motor deficits.

The need for dedicated training for healthcare providers, patients, and caregivers was supported, although by a lower percentage of panelists (67%) as compared to other types of adverse events considered.

Notably, for all the five types of adverse events considered above, the majority of experts agreed (with percentages ranging between 73% for other neurological toxicities and 100% for infections) that there are no significant differences in the occurrence nor in the clinical management of each type of adverse event in patients treated with different anti-BCMA BsAbs agents (i.e., elranatamab, teclistamab, and linvoseltamab).

BsAbs treatment in special patient populations – Additional findings

There was full consensus that no definite factor could currently suggest which specific anti-BCMA BsAbs choose among elranatamab, teclistamab and linvoseltamab across special population subgroups of triple-class exposed RRMM patients.

Supplementary Table S1. Summary of the key considerations on the inpatient management and administration of the step-up dosing phase of anti-BCMA BsAbs in TCE RRMM patients.

Indication	Agreement level (%)	Degree of consensus[1]	Round of agreement
**1. Key procedures to be adopted - Inpatient setting**			
adopt a detailed and comprehensive monitoring protocol, defining roles and responsibilities	100%	Full consensus	Round 1
ensure tocilizumab availability	100%	Full consensus	Round 1
provide training for multi-disciplinary healthcare personnel	93%	Majority consensus	Round 1

Indication	Agreement level (%)	Degree of consensus[1]	Round of agreement
**2. Hospital requirements to start and manage the step-up dosing phase - Inpatient setting**			
Availability of an intensive care unit	90%	Majority consensus	Round 1
Availability of a multidisciplinary team	90%	Majority consensus	Round 1
Availability of specifically trained / highly specialized personnel	90%	Majority consensus	Round 1

[1] Degree of consensus was defined a priori as follows: majority consensus indicated agreement among 66.7%–99.9% of panelists, whereas full consensus indicated unanimous agreement (100%).

Supplementary Table S2. Period of occurrence, prevention, clinical manifestations, and diagnostic workup of treatment-related adverse events associated with anti-BCMA BsAbs in patients with TCE RRMM.

Adverse event	Aspect	Consensus finding	Agreement (%)	Degree of consensus
**Infections**	Period of occurrence	No single phase reached the consensus threshold	—	*No consensus*
**Infections**	Prevention	Prophylaxis recommended	93	Majority consensus
**Infections**	Prevention	Acyclovir and/or trimethoprim–sulfamethoxazole (TMP–SMX; Bactrim)[1]	93	Majority consensus
**Infections**	Prevention	Vaccinations	79	Majority consensus
**Infections**	Immunoglobulin prophylaxis	Recommended	80	Majority consensus
**Infections**	Immunoglobulin prophylaxis	Every 4 weeks/monthly	100	Full consensus
**Infections**	Immunoglobulin prophylaxis	Intravenous administration	67	Majority consensus
**Infections**	Immunoglobulin prophylaxis	For the entire duration of treatment	67	Majority consensus
**Infections**	Main signs and symptoms	Fever	100	Full consensus
**Infections**	Main signs and symptoms	Respiratory distress or other organ-specific symptoms	93	Majority consensus
**Infections**	Diagnostic workup	Chest X-ray and/or CT scan	93	Majority consensus
**Infections**	Diagnostic workup	Blood cultures	80	Majority consensus
**CRS**	Period of occurrence	Step-up dosing phase	93	Majority consensus
**CRS**	Prevention	Inpatients: prophylaxis not recommended	67	Majority consensus
**CRS**	Prevention	Outpatients: prophylactic strategy recommended	93	Majority consensus
**CRS**	Prevention	Outpatients: prophylaxis with tocilizumab may be considered according to local protocols and access conditions[2]	71	Majority consensus
**CRS**	Main signs and symptoms	Fever	100	Full consensus
**CRS**	Main signs and symptoms	Hypotension	87	Majority consensus
**CRS**	Diagnostic workup	Blood cultures and other examinations are required to exclude infection	92	Majority consensus

Adverse event	Aspect	Consensus finding	Agreement (%)	Degree of consensus
**Hematologic toxicities/cytopenia**	Period of occurrence	No single phase reached the consensus threshold	—	*No consensus*
**ICANS**	Period of occurrence	Initial treatment phase/step-up dosing phase	92	Majority consensus
**ICANS**	Prevention	Prophylaxis not recommended	80	Majority consensus
**ICANS**	Main signs and symptoms	Mental confusion	80	Majority consensus
**ICANS**	Main signs and symptoms	Aphasia and/or dysgraphia	67	Majority consensus
**ICANS**	Diagnostic workup	MRI and/or CT scan	87	Majority consensus
**ICANS**	Diagnostic workup	Neurological assessment, including the ICE score	80	Majority consensus
**Other neurologic toxicities**	Period of occurrence	Long-term/late phases of treatment	73	Majority consensus
**Other neurologic toxicities**	Prevention	Prophylaxis not recommended	87	Majority consensus
**Other neurologic toxicities**	Main signs and symptoms	No single sign or symptom reached the consensus threshold	—	*No consensus*
**Other neurologic toxicities**	Diagnostic workup	Specialist neurological examination	73	Majority consensus
**Other neurologic toxicities**	Diagnostic workup	MRI and/or CT scan	67	Majority consensus
**Other neurologic toxicities**	Diagnostic workup	Electromyography	67	Majority consensus

Consensus was prespecified as agreement among ≥66.7% of panelists. Majority consensus indicates agreement among 66.7%–99.9% of panelists, whereas full consensus indicates unanimous agreement (100%). “No consensus” indicates that no single response reached the prespecified threshold. Not all aspects were investigated for all adverse events, and combinations that were not evaluated are not displayed in the Table.

[1] Apart from TMP–SMX use, antibacterial prophylaxis is not generally required at baseline because of the risk of antimicrobial resistance. It is recommended in patients with persistent neutropenia. In patients with grade ≥3 neutropenia, G-CSF is recommended [Ludwig H., et al. Lancet Oncol 2023, 24, e255-e269, doi:10.1016/S1470-2045(23)00159-6].

[2] Feasibility of prophylactic tocilizumab in the outpatient setting may depend on local institutional protocols, drug availability, reimbursement, and authorization for prophylactic off-label use. In Italy, tocilizumab is reimbursed for the treatment of established CRS but not for prophylactic use. The finding should be interpreted as a conditional option, not as a mandatory intervention.

Abbreviations: AE, adverse event; BCMA, B-cell maturation antigen; BsAbs, bispecific antibodies; CRS, cytokine release syndrome; CT, computed tomography; G-CSF, granulocyte colony-stimulating factor; ICANS, immune effector cell-associated neurotoxicity syndrome; ICE, Immune Effector Cell-Associated Encephalopathy; MRI, magnetic resonance imaging; TCE RRMM, triple-class-exposed relapsed/refractory multiple myeloma; TMP–SMX, trimethoprim–sulfamethoxazole.

Supplementary Table S3. Treatment, multidisciplinary management, training, and product-related differences for treatment-related adverse events associated with anti-BCMA BsAbs in patients with TCE RRMM.

Adverse event	Aspect	Consensus finding	Agreement (%)	Degree of consensus
**Infections**	Treatment	Broad-spectrum or antibiogram-guided antibiotic therapy	100	Full consensus
**Infections**	Treatment	Targeted antiviral therapy when an infection is documented	93	Majority consensus
**Infections**	Multidisciplinary management	Multidisciplinary management recommended	100	Full consensus
**Infections**	Multidisciplinary management	Involvement of an infectious disease specialist	100	Full consensus
**Infections**	Training	Training recommended	83	Majority consensus
**Infections**	Training	Patient and caregiver education	80	Majority consensus
**Infections**	Training	Training of infectious disease specialists	67	Majority consensus
**Infections**	Training	Training of General Practitioners	67	Majority consensus
**Infections**	Differences across anti-BCMA BsAbs	No relevant differences identified	100	Full consensus
**CRS**	Treatment	Tocilizumab	93	Majority consensus
**CRS**	Treatment	Dexamethasone or other corticosteroids	87	Majority consensus
**CRS**	Multidisciplinary management	Multidisciplinary management recommended	80	Majority consensus
**CRS**	Multidisciplinary management	Involvement of intensive care specialists in severe cases	83	Majority consensus
**CRS**	Training	Training recommended	87	Majority consensus
**CRS**	Differences across anti-BCMA BsAbs	No relevant differences identified	87	Majority consensus
**Hematologic toxicities/cytopenia**	Treatment	Granulocyte colony-stimulating factor (G-CSF)	100	Full consensus
**Hematologic toxicities/cytopenia**	Differences across anti-BCMA BsAbs	No relevant differences identified	93	Majority consensus
**ICANS**	Treatment	Dexamethasone or other corticosteroids	100	Full consensus
**ICANS**	Multidisciplinary management	Multidisciplinary management recommended	100	Full consensus

Adverse event	Aspect	Consensus finding	Agreement (%)	Degree of consensus
**ICANS**	Multidisciplinary management	Involvement of a neurologist	100	Full consensus
**ICANS**	Training	Training recommended	93	Majority consensus
**ICANS**	Training	Education on general and specific signs of neurotoxicity and use of the ICE score	67	Majority consensus
**ICANS**	Differences across anti-BCMA BsAbs	No relevant differences identified	87	Majority consensus
**Other neurologic toxicities**	Treatment and monitoring	Treatment and long-term monitoring in close coordination with a neurologist	80	Majority consensus
**Other neurologic toxicities**	Multidisciplinary management	Multidisciplinary management recommended	100	Full consensus
**Other neurologic toxicities**	Multidisciplinary management	Involvement of a neurologist	100	Full consensus
**Other neurologic toxicities**	Training	Training recommended	67	Majority consensus
**Other neurologic toxicities**	Differences across anti-BCMA BsAbs	No relevant differences identified	73	Majority consensus

Consensus was prespecified as agreement among ≥66.7% of panelists. Majority consensus indicates agreement among 66.7%–99.9% of panelists, whereas full consensus indicates unanimous agreement (100%). Not all aspects were investigated for all adverse events, and combinations that were not evaluated are not displayed in the Table.

Abbreviations: AE, adverse event; BCMA, B-cell maturation antigen; BsAbs, bispecific antibodies; CRS, cytokine release syndrome; G-CSF, granulocyte colony-stimulating factor; ICANS, immune effector cell-associated neurotoxicity syndrome; ICE, Immune Effector Cell-Associated Encephalopathy; TCE RRMM, triple-class-exposed relapsed/refractory multiple myeloma.
